# YOLO-I3D: Optimizing Inflated 3D Models for Real-Time Human Activity Recognition

**DOI:** 10.3390/jimaging10110269

**Published:** 2024-10-24

**Authors:** Ruikang Luo, Aman Anand, Farhana Zulkernine, Francois Rivest

**Affiliations:** 1 School of Computing, Queen’s University, Kingston, ON K7L 2N8, Canada; ruikang.luo@queensu.ca (R.L.); farhana.zulkernine@queensu.ca (F.Z.); francois.rivest@queensu.ca (F.R.); 2 Royal Military College of Canada, Queen’s University, Kingston, ON K7L 2N8, Canada

**Keywords:** human activity recognition (HAR), Inflated 3D ConvNet (I3D), convolutional neural network (CNN), YOLOv5

## Abstract

Human Activity Recognition (HAR) plays a critical role in applications such as security surveillance and healthcare. However, existing methods, particularly two-stream models like Inflated 3D (I3D), face significant challenges in real-time applications due to their high computational demand, especially from the optical flow branch. In this work, we address these limitations by proposing two major improvements. First, we introduce a lightweight motion information branch that replaces the computationally expensive optical flow component with a lower-resolution RGB input, significantly reducing computation time. Second, we incorporate YOLOv5, an efficient object detector, to further optimize the RGB branch for faster real-time performance. Experimental results on the Kinetics-400 dataset demonstrate that our proposed two-stream I3D Light model improves the original I3D model’s accuracy by 4.13% while reducing computational cost. Additionally, the integration of YOLOv5 into the I3D model enhances accuracy by 1.42%, providing a more efficient solution for real-time HAR tasks.

## 1. Introduction

Human Activity Recognition (HAR) is an important research area with potential applications in various fields such as healthcare, sports, and security [1,2]. Real-time HAR systems have the advantage of being able to recognize and classify human activities in real-time, allowing for immediate feedback or intervention, which is particularly useful in applications such as sports coaching or healthcare monitoring [3,4].

Vision-based HAR systems use visual information to recognize and classify human activities, and provide more accurate and detailed information about the activities being performed compared to wearable sensor-based systems [5]. Video-based HAR can be applied to single-person or crowd activity monitoring, making them suitable for applications such as sports coaching, physical therapy, and surveillance [1,2]. Vision-based real-time HAR systems can provide accurate, detailed, and versatile recognition and classification of human activities in real-time.

With the advancements in computing power, video-based HAR using deep learning has become more performant and useful [6]. It can find extensive applications in areas such as detecting criminal activities and monitoring older people at home or patients in hospitals. Inflated 3D ConvNet (I3D) [7] is one of the successful HAR models. However, real-time applications of I3D are limited by its high computational demand, particularly due to the optical-flow branch. To address this, we introduce YOLO-I3D, which integrates the efficient object detection capabilities of YOLOv5 with I3D to enhance real-time performance while maintaining high accuracy. Additionally, we propose the I3D Light model, focusing on reducing computational complexity while preserving the core strengths of I3D.

After I3D, several other models were proposed such as Separable 3D (S3D-G) [8], TC3D [9], and Depthwise 3D (D3D) [10] to address the high computational cost of traditional 3D convolutional neural networks. S3D and D3D, in particular, reduce the complexity of 3D convolutions by decomposing them into more efficient operations. However, while computationally lighter, these models still require improvements in real-time processing capabilities for deployment in edge devices. In this study, we aim to reduce the computing cost of I3D while maintaining real-time performance and accuracy for real-time applications. Due to the effectiveness of I3D since 2017 in learning spatio-temporal features from videos, the flexibility and compatibility in integrating it into other computing frameworks to create hybrid models capable of processing spatial and temporal information, and the availability of numerous open-source implementations, we opted to use I3D as our base model for video-based HAR.

### Contributions

The following are the key research contributions of this work. We validate our proposed improvements using the Kinetics Human Action Video Dataset [11], which is two orders of magnitude larger than previous datasets, HMDB-51 [12] and UCF-101 [13], with the original I3D model [7].

**Figure 1:**
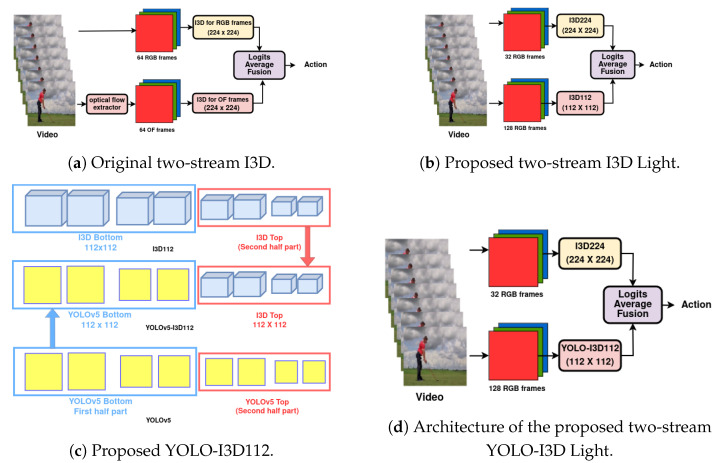
Comparison of Original I3D, Proposed I3D Light, YOLO-I3D112, and two-stream YOLO-I3D Light architectures.

1. We introduce the object detection model, You Look Only Once v5 (YOLOv5) [14], into the RGB branch of the original I3D [7] to construct YOLO-I3D as shown in Figure 1, improving both the efficiency and accuracy of video processing. This upgrade enhances the accuracy of the RGB-only single-branch I3D model by 1.42% on the Kinetics-400 dataset.
2. To reduce the computational cost of the optical flow branch, we replace it with an RGB video processing pipeline to extract simpler motion information. This model, referred to as two-stream I3D Light (Figure 1), improves the original RGB-only I3D accuracy by 4.13% on the Kinetics-400 dataset.
3. Finally, we combine these two enhancements—replacing the RGB branch with YOLOv5 and the optical flow branch with a light motion information processing branch—to create YOLO-I3D Light. The final model, shown in Figure 1, further improves the accuracy of YOLO-I3D by 0.41% on the Kinetics-400 dataset.

The paper is structured as follows: we begin with an introduction to Human Activity Recognition (HAR) and the challenges in achieving real-time performance. Next, we review the related work, highlighting traditional and deep learning-based approaches. Following this, we present our methodology, detailing the YOLO-I3D and two-stream I3D Light models. We then describe the experimental setup and results, comparing the performance of our models with state-of-the-art methods. Finally, we conclude with a discussion of the results and potential directions for future research.

## 2. Related Work

We introduce some preliminary knowledge and discuss the related work in video-based activity recognition along with the common benchmark datasets used in HAR.

As a non-intrusive method, camera-based HAR systems can have extensive applications in areas such as detecting criminal activities, and monitoring older people at home or patients in the hospital [1]. HAR performs a classification task of human activities from static images or videos. We focus specifically on video-based HAR. The recognition part of the process relies on the data features extracted from the videos. Therefore, for accurate classification or HAR, it is critical to extract good data features from the input videos.

### 2.1. Background: Video and Image Feature Extraction

The feature extraction part in many successful HAR models typically applies either (a) image classifiers, which predict a label for the whole image such as Inception-V1 [15], or (b) object detectors, which detect objects within the input image by generating labeled bounding boxes around objects such as YOLOv5 [4]. Modern image classifiers and object detectors usually apply convolutional neural networks (CNNs), which are specifically designed to automatically extract features from spatially distributed multi-dimensional data such as images, instead of performing manual feature engineering. Recent advances in convolutional neural networks (CNNs) for video-based tasks have explored enhanced variations of convolutions to improve feature extraction. For instance, dilated convolutions, which introduce spacing between kernel elements, have been shown to increase the receptive field without a loss in resolution as demonstrated by [16]. Similarly, deformable convolutions, which adapt the shape of the convolution kernel to capture more complex motion patterns, have been successfully applied in tasks like video smoke recognition [17,18]. While these methods focus on improving spatial feature extraction, our work integrates efficient object detection (YOLOv5) with I3D to improve both spatio-temporal feature extraction and real-time performance for HAR tasks.

#### 2.1.1. Image Classification

Image classifiers extract features from the whole image to predict a label. Inception-V1, also known as GoogleNet, is a well-known image classification model. It is a deep convolutional neural network that was developed by Szegedy et al. from Google [15]. The key idea behind the Inception-V1 architecture is to use multiple different sizes of filters in parallel in a single layer, allowing the network to learn both the local and the global features at different scales in an efficient manner. This is achieved by using an “Inception module”, which is a combination of different convolutional filters (1 × 1, 3 × 3, and 5 × 5) and pooling operations that are concatenated together to generate the output of the module. Another key innovation of Inception-V1 is to use 1 × 1 convolutions to reduce the number of input channels to a layer in a way that preserves information as well as reducing computational complexity.

Recent research has incorporated Transformer models to enhance the I3D feature representation capabilities. Guo et al. [19] proposed a Multi-Scale Local Transformer that improves the detection of micro-expressions in long videos by learning multi-scale temporal features. Similarly, Wang et al. [20] introduced a Multi-stream Multi-scale Transformer, improving micro-gesture recognition in long videos by leveraging the attention mechanism of Transformers to capture fine-grained motion patterns. These approaches demonstrate the effectiveness of Transformer models in refining spatio-temporal feature extraction in Human Activity Recognition.

While these works focus on enhancing feature extraction through Transformer models, our approach diverges by integrating the efficient object detection capabilities of YOLOv5 with I3D, optimizing real-time performance for HAR applications.

The 3D CNN [21,22] models serve as powerful methods for extracting features for HAR. The inflated 3D, or I3D, model introduced by Carreira et al. [7] expands the deep 2D CNN architecture of Inception V1 [15] to extract a 3D high-quality spatial appearance and motion features but requires high computational power. Xie et al. [8] proposed a hybrid 2D/3D CNN architecture to balance speed and accuracy for I3D, using low-cost 2D convolutions in the first half of the I3D network. Other 3D CNN models were proposed for object detection as described below.

#### 2.1.2. Object Detection

Object detection is a popular computer vision task that involves detecting and localizing objects within an image or video. One-stage object detectors [14,23,24,25,26] and two-stage object detectors [27,28,29] are two main approaches used in deep learning for object detection [30].

The first two-stage object detection model, Regions with CNN features (R-CNN), was developed by Girshick et al. from Microsoft [27]. In stage one, R-CNN uses selective search to extract region proposals (initial bounding boxes). Selective Search is a region proposal algorithm. At first, it generates a set of region proposals that are likely to contain objects of interest by segmenting an image into regions based on various low-level cues, such as color, texture, and size. Then, it combines these regions into larger ones using a hierarchical grouping strategy, until a set of candidate regions is generated. In stage two, R-CNN computes features for each proposal using CNN and classifies each region by using class-specific linear SVMs. Then, a class-specific bounding-box regressor is used to predict a new bounding box for the detection [27].

One-stage object detectors, such as the YOLO series [14,23,24,25,26], were first proposed by Redmond et al. [23]. They treat object detection (OD) as a regression problem. These models predict object classes and bounding boxes directly from the full image in a single stage without the region proposal. YOLO methods detect anchor boxes as initial bounding boxes. YOLOv5 [14,31] divides the image frame using a grid into multiple cells and assigns three anchor boxes to each cell in every layer of the feature maps for object detection. If the size of input image is 224 × 224, the sizes of three layers of feature maps are 7 × 7, 13 × 13, and 26 × 26. The specific shape and size of the initial anchors are computed from the labels of the training dataset. Because the anchor boxes of one-stage models are computed only once for a given dataset, and the initial boxes of two-stage models are computed for every image, one-stage models are generally faster and simpler than two-stage models. In terms of performance, YOLOv5 (when we designed our methods) achieved state-of-the-art results on various benchmark datasets, such as COCO [32] and Open Images, with high accuracy and fast inference times [4].

### 2.2. Video-Based HAR

The two common methods used for Human Activity Recognition in videos are to use RGB channels in video frames and to use optical flow (OF). RGB frames refer to the traditional approach of using the three-color channels of each frame in a video to recognize actions. OF is a method that calculates the motion vectors of pixels between consecutive frames in a video on the observation image plane. It is used to measure the movement direction and displacement as the instantaneous velocity. The instantaneous velocity of a point is a two-dimensional vector which is called the optical flow vector. In order to compute the OF vector, there are two assumptions: (1) the pixel intensities of an object do not change between the consecutive frames; and (2) the change in time does not cause drastic changes in the object position, that is to say, the displacement between adjacent frames is small. Using these two assumptions, we can obtain the constraint equation as shown in Equation (1), which can be used to compute the OF vector [33]:

(1) I(x,y,t)=I(x+dx,y+dy,t+dt)

where I(x,y,t) is the light intensity of a pixel (x,y) in the first frame, and I(x+dx,y+dy,t+dt) is the light intensity of the same pixel at a new position (x+dx,y+dy) in the second frame after time dt:

(2) $$\frac{\partial I}{\partial x}u+\frac{\partial I}{\partial y}v+\frac{\partial I}{\partial t}=0$$

where $u=\frac{dx}{dt}$ and $v=\frac{dy}{dt}$. By taking the first-order Taylor series approximation of the right-hand side, removing common terms, and dividing by dt, we can obtain Equation (2) to compute optical flow [33].

Zhao et al. showed that both RGB frames and OF have their own advantages and limitations for human action recognition [34]. For instance, RGB frames can capture color and appearance information but may not capture the temporal information and motion patterns that are critical for recognizing some actions.

Chen et al. compared the performance of several methods for human action recognition, including RGB frames, OF, and a combination of the RGB and OF features [35]. The results show that the combination of the RGB and OF features achieves the highest accuracy in recognizing human actions in videos [35].

Researchers have developed many algorithms to estimate the optical flow from RGB frames using Equation (2), such as the Gunnar Farneback algorithm [36] used by OpenCV [37], PWC-Net [38] proposed by Sun et al., and RAFT [39] proposed by Teed et al.

By incorporating both the OF stream and RGB stream in Human Activity Recognition (HAR), a significant improvement in accuracy was achieved [7,40]. The OF stream provides valuable insights into the direction and intensity of object motion across consecutive frames, capturing subtle movements that may not be easily discernible in individual RGB frames. This ability to detect fine-grained motion helps maintain temporal consistency when recognizing and tracking human activities over time, particularly for actions involving continuous or gradual movements. However, it is important to note that estimating OF is computationally demanding. Due to the need for pixel-level correspondences between frames, the process becomes time-consuming. Additionally, OF is sensitive to noise and occlusions within the video sequence, potentially resulting in inaccurate estimations when occlusions or fast motions occur. Moreover, changes in lighting conditions, such as variations in brightness, shadows, or reflections, can affect the accuracy of OF estimation. Consequently, the focus of our work lies in exploring solutions that utilize a simple motion information extraction branch as a replacement for the optical flow branch in the two-stream I3D model.

### 2.3. Datasets for HAR

For the success of HAR, apart from the algorithm, the dataset also plays a very important role. Many researchers and companies have developed a lot of valuable datasets [7,41,42,43,44,45,46,47]. Table 1 shows the main features of commonly used datasets in HAR.

## 3. Methodology

Despite the success, popularity, and compatibility of the two-stream hybrid RGB+OF approach proposed by [7], the high computational cost of the optical flow (OF) branch renders it impractical for real-time applications. In response, this study introduces a three-stage modification strategy to enhance the performance of the model, primarily focusing on time efficiency and reduced computational demand. The subsequent sections detail each stage of improvement and the accompanying validation results.

The original two-stream I3D proposed by [7] has two branches as shown in Figure 1a. The top branch uses RGB frame sequences as input and uses the 3D CNN to extract the spatio-temporal features and classify the activity classes. This single branch can make full use of appearance features but cannot utilize motion features [7]. Therefore, the bottom branch is added, which uses the same 3D CNN structure but with optical flow as the input. The three steps of exploration, development and evaluation of our extensions of the I3D model are illustrated in Section 3.1 to Section 3.3 as summarized below.

### 3.1. Stage 1: Two-Stream I3D Light

The two-stream I3D Light introduces the I3D112 model, which processes lower-resolution, long-range RGB videos to capture motion information, effectively training on temporal features to replace the costly processing of the OF branch in the original I3D model. We apply the following changes to the original I3D model to allow the real-time processing of videos with reduced computing cost while maintaining a comparable accuracy and validate our model using the Kinetics-400 dataset.

1. In order to reduce the training time, we use the pretrained weights of the original model [7,49], and we use 32 RGB frames in the original RGB I3D224 branch instead of 64 frames as used in the published work (following other models such as T-C3D [9], F-E3D [50], and D3D [10]). We denote ***I3D224*** as the original I3D RGB branch which uses 32 frames of images with a resolution of 224 × 224 as input (224 × 224 × 32 instead of 224 × 224 × 64) to extract the spatial features from the data. While reducing resolution can lead to potential information loss, we mitigate this by leveraging pretrained weights from the original I3D model. These weights retain robust spatial feature extraction capabilities learned at a higher resolution, ensuring that essential spatial features are preserved even at a lower resolution. Additionally, we balance spatial and temporal features by combining the lower-resolution motion information branch (112 × 112, 128 frames) with the higher resolution RGB stream (224 × 224, 32 frames), which allows the model to effectively capture both spatial detail and temporal dynamics.
2. We implement a new ***I3D112*** branch to replace the original OF branch in the two-stream I3D model [7]. We denote I3D112 as the motion information branch, which uses 128 frames of images of resolution 112 × 112 as the input for temporal information.
3. As shown in Figure 1b, we combine the top 224 × 224 × 32 RGB I3D224 branch with the bottom 112 × 112 × 128 I3D112 branch to create our two-stream I3D Light model. With I3D112, the proposed I3D Light achieves a balance between maintaining accuracy and reducing computational costs, setting the stage for the subsequent enhancements detailed in the following sections.
4. For studying comparative performance, we train the RGB branch of the original two-stream I3D model and our two-stream I3D Light model using the Kinetics400 dataset. To use the pretrained original I3D weights [7,49] and reduce the training time, we change the pooling size of the last average pooling layer from 7 × 7 to 4 × 4. This ensures that the size of the output feature map from the last average pooling layer matches the input size of that next layer in the I3D model.

### 3.2. Stage 2: YOLO-I3D Model

**Figure 2:**
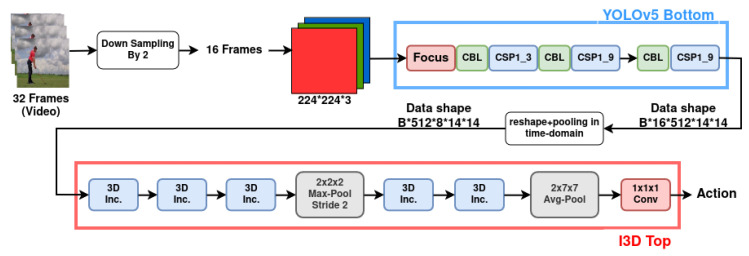
Details of the proposed YOLO-I3D architecture.

One-stage object detectors such as the YOLO models demonstrate superior performance in feature extraction and fast object detection in real-time, which inspired us to use the YOLOv5 model in the hybrid 2D/3D model proposed by [8] to improve the performance of this I3D model. Figure 2 shows our proposed YOLO-I3D model. The changes applied for this extension are as follows.

1. To incorporate the YOLOv5 model in the I3D, we divide the data processing pipeline into the top and bottom parts for both the YOLO (at the bottom) and the I3D112 (top) models. We keep the I3D top part and replace the bottom part with the bottom part of the YOLO model.
2. To make the data sizes compatible, we use an input image resolution of 224 × 224, which generates an output feature map of 512 × 14 × 14 from the YOLO bottom part for each image (512 is channel number and 14 × 14 is the spatial resolution). In order to simulate the effect of a stride size 2 in the first layer of the 3D CNN module in the I3D model, we use a downsampling-by-2 in the data feeding pipeline of YOLO-I3D. During training, the input to YOLO-I3D is a batch of videos (batch size is denoted by B in Figure 2). Each video is sampled to 32 frames of images, and the data shape of the stacked feature maps of YOLO part of YOLO-I3D is B × 16 × 512 × 14 × 14. In order to simulate the pooling layer of the bottom part of the I3D model, we pool the output from the YOLO part of YOLO-I3D. Therefore, the data shape becomes B × 8 × 512 × 14 × 14. In order to match the input data shape of the top part of the I3D model, we switch the order of frame number 8 and the channel dimension 512. So the final shape of the data fed to the top part of I3D model is B × 512 × 8 × 14 × 14.
3. During training, the weights of the YOLO parts of YOLO-I3D are not modified, and only the weights in the top I3D part of YOLO-I3D are optimized. For studying comparative performance, we implement and train the original I3D model I3D224 and our YOLO-I3D model using large Kinetics400 dataset.
4. We fine-tune the original I3D224 model and our YOLO-I3D model on the smaller HMDB51 dataset in order to check the performance of YOLO-I3D using transfer learning. During training, 32 frames of images of resolution 224 × 224 are used as input.

The YOLO-I3D model is much faster than the original I3D model due to the efficiency of YOLOv5 and the architectural modifications made. YOLOv5 is a one-stage object detector optimized for fast inference. It processes entire images in a single forward pass, generating both bounding boxes and classifications simultaneously, thereby reducing the computational overhead compared to two-stage detection methods. Moreover, in YOLO-I3D, the bottom part of the architecture utilizes 2D CNNs, which are computationally less expensive than the 3D CNNs used in the original I3D model. The 2D CNN component processes RGB frames quickly and efficiently, while the 3D CNN component focuses on optimizing temporal features. Since the 2D CNN part is not optimized during training (only the I3D part is fine-tuned), it significantly speeds up both training and inference without sacrificing accuracy.

### 3.3. Stage 3: Two-Stream YOLO-I3D Light

In Section 3.1, we find that replacing the I3D OF branch by a light RGB branch I3D112 can make the computing time per video of two-stream I3D Light 10 times faster than the computing time per video of the original two-stream I3D model. In Section 3.2, we find that replacing the I2D part in the hybrid 2D/3D model proposed by [8] with the feature extractor part of the YOLOv5 model can provide equally good accuracy as the original I3D RGB-only model I3D224, but the training of the resulting new YOLO-I3D is much faster than I3D224. The changes for this extension are explained below.

1. In this section, we put together the two extensions to further enhance the performance of the two-stream I3D Light model. Figure 1c shows the combined YOLO-I3D Light model. The YOLO-I3D model (explained in Section 3.2) is used in the I3D112 branch of the two-stream I3D Light model (explained in Section 3.1). This results in the new two-stream YOLO-I3D Light model containing the YOLO-I3D112 branch as shown in Figure 1d.
2. The overall performance of the model is validated using the Kinetics-400 dataset against the original I3D model.

### 3.4. Model Training and Validation

There are two strategies to fine-tune a model on a small dataset. The first strategy is to freeze the layers other than the last fully connected layer, and train the last layer. Then, the layers are unfrozen and trained together with the last layer. The second strategy is to train all the layers together, which takes more time. We use the first strategy in our experiments because of the small size of the dataset, including the smaller number of classes and corresponding data points.

During fine-tuning, we only train the last fully connected layer of the model leaving all the other layers unchanged. The initial weight values of the unmodified layers use the weights of the pretrained model trained on the Kinetics-400 dataset. The initial weight values of the last layer are assigned random values. Both the RGB224 and the YOLO-I3D112 models in the two branches use 32 frames of images of resolution 224 × 224 as input.

## 4. Implementation

In this section, we describe the implementation details, including the experimental environment, data preprocessing, and model training for the I3D Light, YOLO-I3D, and YOLO-I3D Light models.

### 4.1. Experimental Environment

Our experimental environment is the same for all three stages. The experiment is carried out on a workstation. It has 16 CPU cores (Intel Xeon Gold 6130 CPU @ 2.10 GHz), 64 G memory, and one GPU (NVIDIA Tesla V100 PCIe 32 GB). The OS is Ubuntu 18.04 LTS. The programming language is Python. The deep learning framework is PyTorch [51].

### 4.2. Data Preprocessing

Kinetics-400 [11] and HMDB51 [12] are used for model training and testing to compare with the original I3D model, which is trained and tested on the same datasets. The data are preprocessed before using as described under Section 4. The Kinetics-400 dataset only contains training and validation datasets (no test set). Therefore, we use the validation dataset to report the accuracy as the validation accuracy.

#### 4.2.1. Preprocessing Kinetics-400

Kinetics400 was released by DeepMind in 2017 [11]. It contains 400 human action classes, each of which contains at least 400 video clips. Each clip lasts about 10 s and is taken from different YouTube videos. Since the availability of videos on YouTube changes with time, there are different versions of Kinetics-400. The version of Kinetics-400 we use is from academic-torrents [52]. The dataset from academic-torrents is split into 240,618 training video clips and 19,404 validation video clips. The RGB branch of the original two-stream I3D model is evaluated on the Kinetics-400 dataset. Therefore, we implement and train the RGB branch of the original two-stream I3D model and our two-stream I3D Light model, I3D112, using the Kinetics-400 dataset. We train our stage 2 YOLO-I3D model with this dataset to compare its performance with the original RGB-only I3D model, which was also trained on this dataset. Our stage 3 extension, the YOLO-I3D112 model, is also trained using the Kinetics-400 dataset. While the original I3D and our proposed I3D112 models use 32 frames of images of resolution 224 × 224 as input, the final YOLO-I3D112 model uses 128 frames of images of a lower resolution of 112 × 112 as input to extract better temporal information. The Kinetics-400 dataset is generally well balanced across its 400 action classes, providing a diverse range of human activities for model training and evaluation. However, minor variations in class distribution may occur. Despite this, the large scale of the dataset ensures sufficient samples per class, minimizing the impact of potential class imbalances on the model performance. For training and validating the original RGB I3D224, and our proposed models, we preprocess Kinetics-400 by removing videos with fewer than 128 frames. The final dataset contains 229,516 video clips for training and 18,754 for validation. The videos are converted to jpg images at a resolution of 256 × 256 using OpenCV.

To reduce overfitting, we apply data augmentation by randomly cropping video sequences to specific lengths (32 or 128 frames in the temporal direction and 224 × 224 in the spatial direction) for training. For validation, the sequences are center-cropped. This random cropping makes the training data more diverse, which helps in reducing overfitting.

#### 4.2.2. Preprocessing HMDB51

The HMDB51 dataset [12] is a collection of real videos from various sources, including movies and web videos. We downloaded the HMDB51 dataset from Feichtenhofer’s GitHub repository [22]. The dataset consists of 6766 video clips from 51 action categories, with each category containing at least 101 clips. The minimum clip length of HMDB51 is 1 s [12]. The version of HMDB51 dataset we downloaded has already extracted images from videos with the resolution converted to ensure that the short edge has 256 pixels. We use the split 1 provided in the downloaded dataset which has 3570 training video clips, 1530 validation video clips, and 1666 test video clips. Since the other Kinetics-400 dataset has no test set, we use only the training set and validation set of the HBDB51 dataset. To compare the performance of the fine-tuned YOLO-I3D model with the original RGB-only I3D224 model which was evaluated on the HMDB51 dataset, we fine-tune the original RGB I3D224 and our YOLO-I3D model on HMDB51 dataset using the corresponding models pretrained on the Kinetics-400 dataset. For HMDB51, we remove videos with fewer than 32 frames. The final dataset has 3504 video clips for training and 1251 for validation. The preprocessing involves scaling images to 256 × 256 and cropping to 224 × 224. For temporal feature extraction, we crop image sequences to 32 frames for training and center-crop them for validation.

### 4.3. Model Training Using Sub-Epoch and Sub-Dataset

Since Kinetics-400 is very big, each training epoch takes about 3 and half hours to train a single branch I3D224 or I3D112 model. In order to check the effect of hyper-parameter optimization and avoid wasting too much time with the wrong hyper-parameters, we propose sub-epoch-based training instead of ordinary epoch-based training as explained below.

***Algorithm: Sub-epoch runner***

1. Step 1: Loading and preprocessing data.
  - Load training dataset and validation dataset.
  - Divide the whole training dataset into *N* (such as 10) subsets randomly with equal probability. Keep the validation dataset undivided.
  - Create *N* sub-data-loaders corresponding to the *N* sub-datasets for training and one data loader for the validation dataset.
2. Step 2: Training.
  - Divide each epoch into *N* sub-epochs. Each sub-epoch performs training on the corresponding sub-dataset and a validation on the whole validation dataset.
  - If the validation accuracy does not improve after a specified number of training epochs, the learning rate is decreased based on a given schedule, and the model weights are set to the values providing the best validation accuracy until that time point.
3. Step 3: Stop the training if the epoch number reaches a specified value or the early stop condition is satisfied. Otherwise, go back to Step 2.
  - We divide each epoch into *N* sub-epochs in all the experiments in this section. The learning rate is decreased in the sub-epoch runner when there is no improvement in the validation accuracy after *N* sub-epochs (i.e., one entire epoch).

### 4.4. Model Training

In this section, we first explain the general information that applies to all three models proposed in this work. Any custom processing for specific models is explained under the respective subsection.

***Model Codebases:*** Miracleyoo’s PyTorch implementation [49] of I3D is used as the code base. The code of Bottom-YOLOv5 is modified from the official YOLOv5 GitHub repository [14].

***Initial Weights:*** We use the weights of the original I3D [7] model trained on the Kinetics-400 dataset as the initial weights of I3D224 and I3D112 to save training time. These weights are imported from the original TensorFlow version of the I3D pretrained weights on Kinetics-400 [7,53].

***Parameter Optimization:*** For all the models, with the initial weights set as mentioned above, we train the models on the big Kinetics-400 datasets using the sub-epoch training strategy to determine the optimal hyper-parameter values. When training with sub-epochs and the Kinetics dataset, validation is performed on the whole validation dataset at the end of each sub-epoch. If the validation accuracy does not improve after a predefined number of sub-epochs, which is equivalent to training on the whole training dataset, the learning rate is changed to the next one from a predefined list of decreasing learning rates. At the same time, the model weights are set to the weights that have provided the best accuracy achieved so far.

For the stage-1 I3D Light model, SGD optimizer is used during training on the Kinetics data with a list of gradually decreasing learning rates.

For the stage-2 YOLO-I3D and the RGB224 models, we use the SGD optimizer for training on the Kinetics dataset. After training on the Kinetics dataset, we fine-tune the stage-2 YOLO-I3D and the original RGB224 models on the HMDB51 dataset. We find that Adam optimizer works better than SGD optimizer for the HMDB51 dataset.

For training YOLO-I3D with the Adam optimizer, the super parameter weight decay is set to 1.0 $\times \;10^{-4}$ and the initial learning rate is set to 1.0 $\times \;10^{-3}$. For I3D224, if the validation accuracy has not improved after 10 epochs, the learning rate is divided by 10. For YOLO-I3D, if the validation accuracy has not improved after 5 epochs, the learning rate is divided by 10. At the same time, the model weights are set to the weights corresponding to the best accuracy achieved so far.

After finishing the first-round of training of YOLO-I3D, a second-round of training is performed with different hyper-parameters. For I3D224, the super parameter weight decay is set as 1.0 $\times \;10^{-4}$, and the initial learning rate is set as 1.0 $\times \;10^{-5}$. If the validation accuracy has not improved after 10 epochs, the learning rate is divided by 10. For YOLO-I3D, the super parameter weight decay is set as 1.0 $\times \;10^{-4}$, and the initial learning rate is set as 1.0 $\times \;10^{-4}$. If the validation accuracy has not improved after 5 epochs, the learning rate is divided by 10. At the same time, the model weights are set to the weights corresponding to the best accuracy achieved so far.

To train the stage-3 two-stream YOLO-I3D Light, we use the fixed sub-epoch runner and divide each epoch into five sub-epochs with the Adam optimizer. The learning rate is divided by 10 if no improvement in validation accuracy is achieved after five sub-epochs (i.e., one entire epoch). At the same time, the model weights are set to the weights corresponding to the best accuracy achieved so far.

***Loss Function:*** HAR is a multi-class classification problem. Therefore, we use the multi-class cross-entropy loss function in PyTorch [54] as shown in Equation (3) [55]:

(3) $$L\left(\hat{y},y\right)=-\sum_{k=1}^{K}y_{k}\log{\hat{y}}_{k}$$

where $y_{k}$ is the true label having a value 0 or 1, indicating whether class *k* is the correct classification, and ${\hat{y}}_{k}$ is the prediction. For one sample video, the class with the smallest loss is the predicted class.

***Evaluation Criteria:*** For the HAR classification problem, accuracy is used as the validation criterion as shown in Equation (3). The models are evaluated with the validation dataset, and the results report the accuracy on the validation set for comparative analysis. Accuracy is chosen as the primary evaluation metric for this study due to the balanced nature of the Kinetics-400 dataset, where each class has a relatively similar number of instances. Accuracy provides a straightforward measure of performance in such balanced datasets. While other metrics such as F1-score, precision, or recall may offer additional insights, accuracy remains a standard evaluation metric for large-scale balanced datasets like Kinetics-400. For smaller or imbalanced datasets, future work may incorporate additional metrics to better capture model performance:

(4) $$\mathrm{Accuracy}=\frac{N_{\mathrm{correct}}}{N_{\mathrm{total}}}$$

where $N_{\mathrm{correct}}$ is the number of samples that were correctly predicted by the model, and $N_{\mathrm{total}}$ is the total number of samples in the validation dataset.

## 5. Validation

We conduct multiple experiments to explore how each change to the I3D model affects its performance and compare with the original I3D model as illustrated below.

### 5.1. Experiment 1: Two-Stream I3D Light

We evaluate the performance of the proposed I3D112 model by comparing it with the original I3D RGB I3D224 model in a single-stream scenario. Next, we train the two-stream I3D Light model which has the I3D224 and I3D112, where the latter replaces the OF branch. After I3D224 and I3D112 are trained separately, we train the combined two-stream I3D Light model composed of the I3D224 and I3D112 model.

Accuracy computing resources and prediction times are used as the comparison criteria since the objective is to achieve comparable accuracy with reduced computing resources for real-time applications.

#### 5.1.1. Model Accuracy

**Figure 3:**
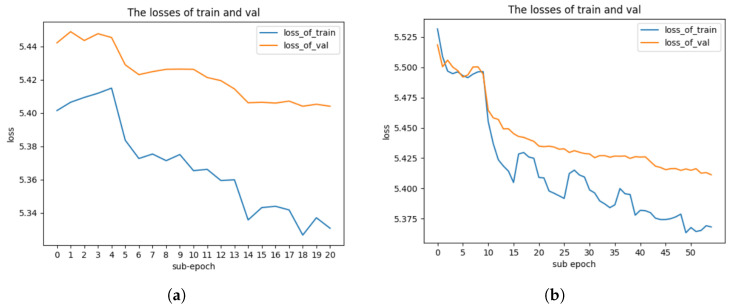
Comparison of loss graphs for I3D224 and I3D112 branches on Kinetics-400 using sub-epoch where N = 10. (**a**) Loss graph of I3D224 branch on Kinetics-400 using sub-epoch where N = 10. (**b**) Loss graph of I3D112 branch on Kinetics-400 using sub-epoch where N = 10.

***I3D224:*** When training the I3D224 model, we use [0.008, 0.004, 0.002, 0.0008, 0.0004, 0.0002] as a sequence of learning rates. The loss graph of 20 sub-epochs is shown in Figure 3a. The accuracy of the I3D224 model is shown in Table 2.

***I3D112:*** To train I3D112, we use [0.01, 0.001, 0.0008, 0.0004, 0.0002] as the list of learning rates. The loss graph of 55 sub-epochs is shown in Figure 3b. The accuracy of I3D112 is shown in Table 2. The results in Table 2 show that when the I3D224 and I3D112 branches are trained together (with combined tuning), the training accuracy is lower, but the validation accuracy is higher compared to when the branches are tuned separately (no combined tuning). This phenomenon can be explained by the regularization effect that occurs during combined tuning. By training both branches simultaneously, the model avoids overfitting to the training data and instead learns more generalized features that benefit performance on unseen data. Although this may result in a slight decrease in training accuracy, the model is better equipped to generalize, which leads to an improvement in validation accuracy. Additionally, the complexity of the optimization process increases when tuning both branches together, making it harder for the model to achieve high training accuracy. However, this complexity contributes to learning more robust features across both the spatial and temporal domains, ultimately enhancing the model’s generalization ability.

**Figure 4:**
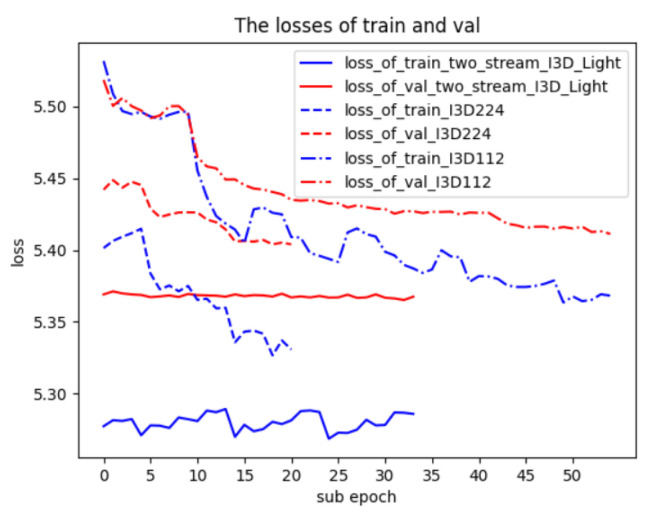
Loss graphs of two-stream I3D Light, I3D224, and I3D112 on Kinetics-400 using sub-epoch where N = 10.

***Two-stream I3D Light:*** We apply a simple fusion method as used in the original two-stream I3D to combine the RGB I3D224 and the I3D112 models into a two-stream I3D model. Outputs from I3D224 and I3D112 are averaged before the softmax function is applied. The accuracy of the proposed two-stream I3D Light is shown in Table 2. In order to improve the accuracy further, we train the I3D224 branch and I3D112 branch together as a two-stream model. The loss graphs of 33 sub-epochs are shown in Figure 4. We also show the loss graphs of I3D224 and I3D112 in Figure 4.

As shown in Figure 4, the training loss and validation loss of two-stream I3D Light are relatively small (solid lines), and the variation in the training loss and validation loss of two-stream I3D Light varies within a small range. The reason is that the initial weights of I3D224 and I3D112 are close to the final optimal values of two-stream I3D Light model during the combined training of two branches in two-stream I3D Light.

The accuracy after combined tuning of I3D224 + I3D112 is shown in Table 2.

#### 5.1.2. Effectiveness of Sub-Epoch Training

To validate that the sub-epoch training does not affect the accuracy significantly, we train the models on the whole training dataset without dividing it into sub-datasets. We observe very little improvement in training the model without sub-epoch training. Table 3 includes the final results of I3D224, I3D112, and two-stream I3D Light after sub-epoch training and two further regular epoch training rounds. As a comparison, the test accuracy of the original I3D on miniKinetics [7] is also shown in Table 3.

#### 5.1.3. Computational Cost

In order to compare the computing resources required by two-stream I3D Light, we use a Python package torchinfo [56] to estimate the total memory size and the total number of addition and multiplication operations required by the PyTorch model. The estimated values as shown in Table 4 are very close for I3D224 and I3D112. We also compute the average prediction or scoring times for I3D224 and I3D112 on our system by using 100 predictions on one video. It shows very similar performance of 0.028(s) when rounded to the third digit.

#### 5.1.4. Discussion

There is a notable difference between the results of the original I3D [7] on miniKinetics and our results on the Kinetics-400 dataset as shown in Table 2. Several factors may contribute to this. First, the original I3D uses 64 frames, while our I3D224 model uses 32 frames. Second, miniKinetics contains fewer classes (213) compared to Kinetics-400, which may lead to better accuracy on smaller datasets. Third, due to resource constraints, our models are trained for fewer epochs than the original I3D. Nevertheless, for consistency, we train both I3D224 and I3D112 under the same conditions. However, the accuracy improvements seen when combining YOLO-I3D with I3D Light are relatively small (1.42% accuracy improvement on Kinetics-400). This marginal improvement is primarily because YOLOv5 efficiently extracts features in the RGB branch, which complements I3D but does not drastically change the performance of the model when used in combination with I3D Light, which already incorporates efficient motion information processing. The main advantage of this integration lies in the computational efficiency and the model’s ability to operate in real-time scenarios without a significant trade-off in accuracy. As shown in Table 4, the execution time for I3D112 is significantly lower than that of traditional Optical Flow (OF) methods, making it a viable option for real-time applications, especially on edge devices. Specifically, I3D112 executes 20 times faster than the Gunnar Farneback and PWC-Net algorithms and is about 14 times faster than the RAFT algorithm. Although OF could be precomputed for training, it poses a bottleneck in real-time deployments. Our two-stream I3D Light model addresses this limitation by providing a much faster alternative while maintaining competitive accuracy, thus validating its effectiveness in real-time applications.

### 5.2. Experiment 2: YOLO-I3D

We evaluate YOLO-I3D in two steps. First, we compare the performance of the proposed YOLO-I3D with the original I3D224 on the Kinetics400 dataset. Then, we apply transfer learning to fine-tune the model on a small dataset HMDB51 and again compare the above two model performances.

#### 5.2.1. Model Performance

**Figure 5:**
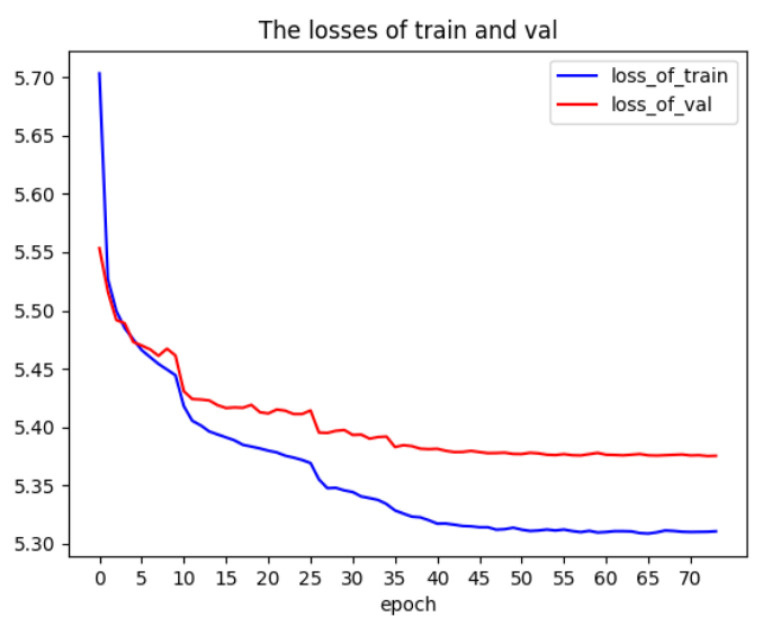
Loss graph of YOLO-I3D on dataset Kinetics-400.

***Using Kinetics-400:*** During the training of YOLO-I3D on Kinetics-400, we decrease the learning rates based on this list: [8.0 $\times \;10^{-2}$, 4.0 $\times \;10^{-2}$, 2.0 $\times \;10^{-2}$, 8.0 $\times \;10^{-3}$, 4.0 $\times \;10^{-3}$, 2.0 $\times \;10^{-3}$, 1.0 $\times \;10^{-3}$]. We use the weights of the top part Figure 2 of the original I3D [37] to initialize the weights of TOP-3D CNN part of YOLO-I3D. In total, 72 epochs are run during the training of YOLO-I3D on Kinetics-400. The loss graph of the 72 epochs is shown in Figure 5. The final accuracy of YOLO-I3D on Kinetics-400 is shown in Table 5. As shown in the table, YOLO-I3D can improve the accuracy of I3D224 by 1.42%, and the training time of YOLO-I3D is less than half the training time of I3D224.

**Figure 6:**
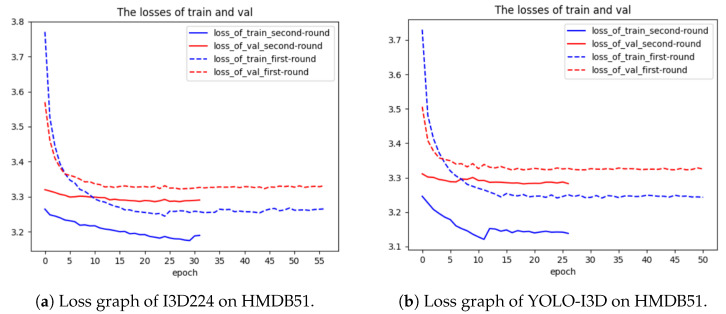
Comparison of loss graphs for I3D224 and YOLO-I3D on dataset HMDB51.

***Using HMDB51:*** The loss charts from the first and second rounds of training when fine-tuning I3D224 on the HMDB51 dataset are shown in Figure 6a. The decrease in training loss and validation loss is very fast and smooth during the first-round training, which shows that the performance of fine-tuning of I3D224 is good.

When fine-tuning YOLO-I3D on the HMDB51 dataset, the loss charts from the first and second rounds of training are shown in Figure 6b. The decrease in loss is very fast and smooth during the first-round training, which shows that the performance of the fine-tuning of YOLO-I3D is as good as I3D224.

#### 5.2.2. Discussion

There are two reasons that YOLO-I3D achieves improvements over I3D224 in execution time and accuracy as shown in Table 5 with the Kinetics-400 dataset: (1) the YOLO part of the YOLO-I3D model uses 2D CNN instead of 3D CNN, which is used in the I3D224 model, and (2) the YOLO part does not take part in the optimization during training.

The accuracy of YOLO-I3D after fine-tuning on the small HMDB51 dataset is shown in Table 6. We also include the baseline result from Xu et al. [57], which reports a validation accuracy of 67.9% on the HMDB51 dataset. Our results demonstrate that both YOLO-I3D and I3D224 outperform this baseline, achieving a validation accuracy of 70.98%. Additionally, YOLO-I3D shows a higher training accuracy (84.64%) compared to I3D224 (79.71%), indicating a higher degree of overfitting on this small dataset. This overfitting may be due to the hybrid 2D/3D architecture of YOLO-I3D, which introduces some mismatch between the 2D CNN and 3D CNN components when trained on a smaller dataset like HMDB51. The inclusion of Xu et al.’s result provides a meaningful comparison to validate the efficacy of our method against a fast human action recognition network.

While our proposed YOLO-I3D model demonstrates improved performance across various datasets, there are some limitations when applied to smaller datasets such as HMDB51. One challenge with HMDB51 is the relatively small number of video clips and shorter average video lengths, which can make it difficult for models to capture complex motion patterns compared to larger datasets like Kinetics-400. Additionally, the diversity of actions in HMDB51 is limited, potentially leading to overfitting in models with higher parameter counts. We recognize that these limitations may impact the generalization ability of our model, and future work will focus on exploring techniques such as data augmentation or transfer learning to better address these challenges.

### 5.3. Experiment-3: Two-Stream YOLO-I3D Light

First, we test the effectiveness of the proposed YOLO-I3D112 in the original two-stream I3D model. Next, we test the 2-stream YOLO-I3D Light model, which has I3D224 as stream one and YOLO-I3D112 as stream two.

#### 5.3.1. Model Accuracy

**Figure 7:**
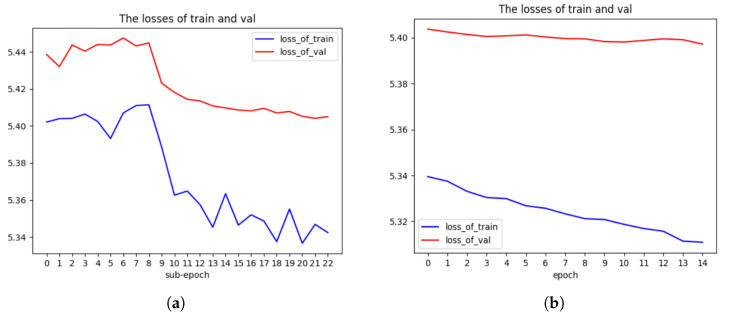
Comparison of loss graphs for YOLO-I3D112 on Kinetics-400 using and not using sub-epoch. (**a**) Loss graph of YOLO-I3D112 on Kinetics-400 using sub-epoch. (**b**) Loss graph of YOLO-I3D112 on Kinetics-400 not using sub-epoch and on the full dataset.

***I3D112 branch:*** First, we train YOLO-I3D112 by using sub-epoch method. The super parameter weight decay is set as 1.0 $\times \;10^{-5}$, and the initial learning rate is set as 1.0 $\times \;10^{-4}$. If the validation accuracy is not improved after five sub-epochs, the learning rate is divided by 10. At the same time, the model weights are set to the weights corresponding to the best accuracy achieved so far. The weights of YOLO-I3D trained on Kinetics-400 are used as the initial values of weights of YOLO-I3D112. We run 22 sub-epochs during this training. The loss graph of YOLO-I3D112 is shown in Figure 7a.

Next, in order to improve the accuracy further, the model is trained on the whole training dataset using ordinary epoch training for 14 epochs. The loss graph of YOLO-I3D112 is shown in Figure 7b. The accuracy of YOLO-I3D112 is shown in Table 7. As a comparison, the result of I3D112 is also shown in Table 7. As shown in Figure 7b, the improvement in loss after training on the whole training set is better than that of I3D112 as presented in Section 5.1.1. The reason may be that we only train two epochs for the whole training set. If we train I3D112 for more than 10 epochs on the whole training set, we may be able to find an improvement similar to that shown in Figure 7b. As shown in Table 7, YOLO-I3D112 can increase the accuracy of I3D112 on Kinetics-400 by 1.44%. So, it is effective to combine YOLOv5 with I3D112 to improve the accuracy of a single branch.

***Two-Stream YOLO-I3D Light:*** After we finish training I3D224 branch and YOLO-I3D112 branch separately, we test the performance of two-stream YOLO-I3D Light (i.e., I3D224 + YOLO-I3D112). During the training of the two-stream YOLO-I3D Light, we use the weights of I3D224 and YOLO-I3D112 obtained before as the initial values of two-stream YOLO-I3D Light and find that there is no improvement in accuracy. The reason may be that the weights of the I3D224 branch and YOLO-I3D112 branch trained separately before are very close to the optimal values of the weights of the two-stream YOLO-I3D Light. We therefore train the two-stream YOLO-I3D Light for only two epochs with the full dataset.

The accuracy of the proposed two-stream YOLO-I3D Light is shown in Table 7. As a comparison, the result of the two-stream I3D Light (non-YOLO) is also shown in Table 7. It can be seen that two-stream YOLO-I3D Light only increases the accuracy of the two-stream I3D Light by 0.41%, but YOLO-I3D can increase the accuracy of I3D224 by 1.42% on the Kinetics-400 dataset (in Table 5). The reason may be that YOLO is good at extracting the appearance features and not so good at extracting the motion features.

#### 5.3.2. Execution Time

For comparison, the execution times of every branch used in the two-stream original I3D, two-stream I3D Light, and two-stream YOLO-I3D Light on Kinetics-400 datasets are shown in Table 8. The comparison of the training and validation times per epoch are also shown in Table 8.

#### 5.3.3. Discussion

The execution time per video is similar for YOLO-I3D112, I3D112, YOLO-I3D, and I3D224. The training time per epoch for YOLO-I3D and YOLO-I3D112 is about half the training time per epoch compared to I3D224 and I3D112. The reason is that the first half part of the YOLO-I3D model and YOLO-I3D112 model uses 2D CNN instead of 3D CNN as the one in the I3D224 model and I3D112 model, and the weights of the 2D-CNN part from YOLOv5 remain unchanged and do not take part in the optimization during training. The validation time is not so clear because the data loading time has a big effect in the validation phase.

## 6. Conclusions

Real-time Human Activity Recognition (HAR) is essential in fields like healthcare and sports. Traditional high-accuracy models like two-stream I3D proposed by [7] are not suited for real-time use due to the heavy computational demands of the optical flow components. This paper presents a solution by introducing a lightweight motion feature extractor, I3D112, to replace the optical flow branch in the two-stream I3D model. The two-stream I3D Light model combines the proposed I3D112 branch with the I3D224 branch and enhances the accuracy of I3D224 by 4.13% (Table 3) on the Kinetics-400 dataset. The proposed I3D112 model has a similar execution time to the I3D224 branch (Table 4), making real-time application possible. Further, we develop YOLO-I3D, which outperforms the original I3D224 model by 1.44% in accuracy (Table 5) and increases training speed. Combining these methods, the proposed two-stream YOLO-I3D Light model improves accuracy (Table 7) and significantly reduces training time (Table 8), proving its effectiveness for real-time HAR systems deployment.

For future research, additional evaluations of YOLO-I3D are warranted, especially given its limitations for smaller datasets like HMDB51, where the model may overfit due to the smaller number of video clips and limited diversity. Testing on other datasets like UCF101 and miniKinetics could better demonstrate the model’s robustness across various activities. Exploring the integration of YOLO with Graph Convolutional Networks (GCNs) is another promising direction, as GCNs can process the heterogeneous data provided by YOLO object detection, enriching the action recognition process, particularly for activities involving object interactions.

## Figures and Tables

**Table 1:** Popular datasets used in HAR.

Dataset	Year	Frame Rate	Number of Actions	Number of Videos	Average Clip Length	Video Resolution
KTH [42]	2004	25 FPS	6	2391	4 s	160 × 120
HMDB51 [12]	2011	30 FPS	51	6849	2.5 s	340 × 256 *
UCF101 [13]	2012	25 FPS	101	13,320	7.2 s	320 × 240
Sports-1M [43]	2014	variable	487	1,000,000	5 m 36 s	variable
Charades [44]	2015	24 FPS	157	9848	30 s	640 × 480
NTU RGB+D [45]	2016	30 FPS	60	56,880	8.6 s	512 × 424
miniKinetics [7]	2017	variable	213	120,000	10 s	variable
Kinetics-400 [11]	2017	variable	400	306,245	10 s	variable
Something-Something V1 [46]	2017	24 FPS	174	108,499	4.0 s	84 × 84
Something-Something V2 [46]	2018	24 FPS	174	220,847	4.0 s	84 × 84
Kinetics-600 [47]	2018	variable	600	495,547	10 s	variable
Kinetics-700 [48]	2019	variable	700	650,317	10 s	variable
NTU RGB+D 120 [41]	2019	30 FPS	120	114,480	8.5 s	512 × 424

Note: * means most of the videos have a resolution of 340 × 256 with some variation.

**Table 2:** Results of the two-stream I3D Light on Kinetics-400 dataset using sub-epoch training strategy.

Model	Input	Training Accuracy	Validation Accuracy
I3D224	32 frames	69.84%	61.00%
I3D112	128 frames	65.89%	60.02%
I3D224+I3D112 (No combined tuning)	32 frames + 128 frames	80.15%	64.47%
I3D224+I3D112 (With combined tuning)	32 frames + 128 frames	75.51%	65.09%

**Table 3:** Final results of two-stream I3D Light on dataset Kinetics-400 without sub-epoch.

Model	Input	Training Accuracy	Validation Accuracy
I3D224 only	224 × 224 × 32	69.65%	61.03%
I3D112 only	112 × 112 × 128	66.91%	60.39%
Two-stream I3D224 + I3D112 (With combined tuning)	224 × 224 × 32 + 112 × 112 × 128	76.20%	65.16%
Original I3D RGB branch only on miniKinetics * [7]	224 × 224 × 64	N/A	74.1% (test accuracy)
Original two-stream I3D on miniKinetics * RGB branch+OF branch [8]	224 × 224 × 64 + 224 × 224 × 64	N/A	78.7% (test accuracy)

Note: * miniKinetics is a smaller dataset than the full Kinetics-400. It has only 213 classes with a total of 120 k clips [7].

**Table 4:** Comparison of computing resources needed by two-stream I3D Light.

Model	Estimated Total Memory Size	Total Mult-Adds	Avg. Execution Time per Video	Training Time per Epoch	Validation Time per Epoch
I3D112 only	12,490.13 MB	944.80 G	0.028 s	3 h 24 m 3 s	7 m 22 s
I3D224 only	12,528.13 MB	949.73 G	0.028 s	3 h 48 m 43 s	13 m 10 s
Two-stream I3D224 + I3D112 [8]	25,018.26 MB	1894.53 G	0.056 s	N/A	N/A
OF (Farneback) only [36]	N/A	N/A	0.638 s	N/A	N/A
Two-stream I3D224 + OF (Farneback) [36]	N/A	N/A	0.666 s	N/A	N/A
OF (PWC-Net) only [38]	N/A	N/A	0.574 s	N/A	N/A
Two-stream I3D224 + OF (PWC-Net) [38]	N/A	N/A	0.600 s	N/A	N/A
OF (RAFT) only [39]	N/A	N/A	0.390 s	N/A	N/A
Two-stream I3D224 + OF (RAFT) [39]	N/A	N/A	0.418s	N/A	N/A

**Table 5:** Results of YOLO-I3D on dataset Kinetics-400.

Model	Input	Training Accuracy	Validation Accuracy	Training Time per Epoch	Validation Time per Epoch
YOLO-I3D	32 frames	69.05%	62.42%	1 h 16 m 37 s	5 m 02 s
I3D224	32 frames	69.84%	61.00%	3 h 24 m 03 s	7 m 22 s

**Table 6:** Results of YOLO-I3D and I3D224 on dataset HMDB51. Bold numbers represent the best.

Model	Input	Training Accuracy	Validation Accuracy
Xu et al. [57] (Baseline)	-	-	67.9
YOLO-I3D	32 frames	84.64%	**70.98%**
I3D224	32 frames	79.71%	**70.98%**

**Table 7:** Final results of two-stream YOLO-I3D Light and two-stream I3D Light on dataset Kinetics-400.

Model	Input	Training Accuracy	Validation Accuracy
YOLO-I3D112	128 frames	71.52%	61.46%
I3D112	128 frames	65.89%	60.02%
Two-stream YOLO-I3D Light I3D224 + YOLO-I3D112	32 frames + 128 frames	N/A	65.57%
Two-stream I3D Light I3D224 + I3D112	32 frames + 128 frames	76.20%	65.16%

**Table 8:** Comparing execution time of proposed models and original I3D.

Model	Average Execution Time per Video	Training Time per Epoch	Validation Time per Epoch
YOLO-I3D112	0.024 s	1 h 26 m 29 s	6 m 30 s
I3D112	0.028 s	3 h 48 m 43 s	13 m 10 s
YOLO-I3D	0.024 s	1 h 16 m 37 s	5 m 02 s
I3D224	0.028 s	3 h 24 m 03 s	7 m 22 s
Two-stream I3D Light	0.056 s	7 h 22 m 27 s	16 m 14 s
Two-stream YOLO-I3D Light	0.052 s	5 h 19 m 39 s	13 m 34 s
Optical flow	0.638s	N/A	N/A
Original two-stream I3D	0.666 s	N/A	N/A

## Data Availability

We have used publicly available datasets in this research.
